# Comparison of molecular detection methods for pertussis in children during a state-wide outbreak

**DOI:** 10.1186/s12941-016-0142-4

**Published:** 2016-04-27

**Authors:** X. Qin, D. M. Zerr, M. P. Kronman, A. L. Adler, J. E. Berry, S. Rich, A. M. Buccat, M. Xu, J. A. Englund

**Affiliations:** Microbiology Laboratory, Seattle Children’s Hospital, Seattle, WA USA; Department of Laboratory Medicine, Seattle Children’s Hospital, University of Washington, Seattle, WA USA; Division of Pediatric Infectious Diseases, Department of Pediatrics, Seattle Children’s Hospital, University of Washington, Seattle, WA USA

**Keywords:** Respiratory viruses, *Bordetella pertussis*, Rapid diagnosis

## Abstract

A state-wide pertussis outbreak occurred in Washington during the winter–spring months of 2012, concurrent with respiratory viral season. We compared performance characteristics of a laboratory-developed pertussis PCR (LD-PCR for *Bordetella pertussis*, *Bordetella parapertussis*, and *Bordetella holmesii*) and rapid multiplex PCR (RM-PCR) for respiratory viruses (FilmArray™, BioFire, *B. pertussis* data unblinded following FDA approval post outbreak). We analyzed three cohorts of patients using physician testing orders as a proxy for clinical suspicion for pertussis or respiratory viruses: Cohort 1, tested by LD-PCR for pertussis pathogens only by nasopharyngeal swab; Cohort 2, by RM-PCR for respiratory viruses only by mid-nasal turbinate swab; and Cohort 3, by both methods. *B. pertussis* was detected in a total of 25 of the 490 patients in Cohort 3 in which LD-PCR detected 20/25 (80 %) cases and the RM-PCR detected 24/25 (96 %; p = 0.2). Pertussis pathogens were detected in 21/584 (3.6 %) of samples from Cohort 1 where clinicians had a relatively strong suspicion for pertussis. In contrast, *B. pertussis* was detected in only 4/3071 (0.1 %) specimens from Cohort 2 where suspicion for pertussis was lower (p < 0.001 for comparison with Cohort 1). In summary, the two laboratory methods were comparable for the detection of *B. pertussis*.

## Background

Resurgence of pertussis disease has been documented in the United States [1]. Nearly 5000 *Bordetella pertussis* cases were reported in Washington State in 2012, the highest in 70 years [2, 3]. During respiratory seasons, clinical differentiation of pertussis from viral pathogens is problematic in pediatric populations, especially in young children [4, 5]. Identification of pertussis pathogens—as well as other agents causing respiratory and cough illnesses—has important implications for patient care, vaccination recommendations, antimicrobial stewardship, and implementation of appropriate hospital—and community-based infection prevention.

Historically, laboratory diagnosis of pertussis and respiratory viral pathogens required technically demanding methods maintained by specialized laboratories. Polymerase-chain-reaction (PCR) has greatly improved test sensitivity and reduced the time-to-result, enabling pathogen detection in patients long after symptom onset or antibiotic therapy [6]. However, most PCR tests provide high throughput capacity but not rapid real-time test results due to the common practice of batching samples to gain efficiency [7, 8]. Moreover, laboratory diagnosis of respiratory infections has been affected by substantial variations in collection and transport methods. The emergence of US Food and Drug Administration (FDA)-approved automated, rapid “mega”-multiplex PCR platforms (RM-PCR) such FilmArray™ (BioFire, Salt Lake City, UT) exemplifies a technical breakthrough, enabling rapid diagnosis of acute viral and bacterial respiratory infections from a swab specimen and a single molecular panel.

**Fig. 1:**
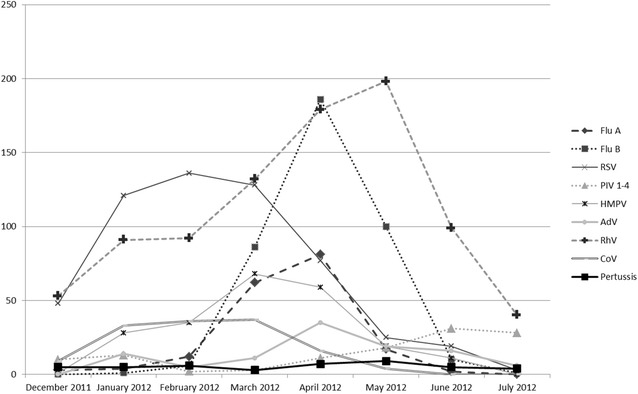
Number of laboratory reported pertussis and respiratory viral infections during the 7.5 month study period (December 14, 2011–July 31, 2012)

The well-recognized 2012 pertussis outbreak in Washington State overlapped a respiratory viral season characterized by wide-spread circulation of influenza, respiratory syncytial virus (RSV), and other respiratory viruses (Fig. 1) [7, 9]. Both RM-PCR (FilmArray) for respiratory viruses and a laboratory-developed PCR (LD-PCR) for three pertussis pathogens (*B. pertussis*, *B. parapertussis*, and *B. holmesii*) were deployed during this outbreak [8]. At the time, detection of *B. pertussis* was included within the panel of RM-PCR panel but results were unavailable to laboratory for reporting pending FDA approval. *B. pertussis* detection results were obtained post hoc following licensure of the expanded RM-PCR panel by FDA in October 2012 [10]. In addition to increased laboratory utilization of high throughput LD-PCR during the pertussis outbreak, our diagnostic operation also included culture for all PCR-positive samples, achieving a 48 % culture-positivity rate that was useful in statewide epidemiological studies of pertussis [3].

In this report, we compare results from our in-house LD-PCR—which enabled batching and high throughput—with those from the rapid multiplex RM-PCR assay. We defined the performance characteristics of these two different approaches in identifying pertussis pathogens and analyzed the utility of RM-PCR during the overlapping viral respiratory season in the pediatric urgent care and hospital-based setting.

## Methods

### Overview and specimen inclusion criteria

The protocol for this retrospective cohort study was approved by Seattle Children’s Institutional Review Board. All authors declare no competing interests that could potentially influence data analysis and conclusions.

We analyzed specimens collected from patients aged from birth to 21 years at Seattle Children’s Hospital from December 14, 2011 (the date of RM-PCR implementation replacing all other methods for respiratory viral diagnosis prior to the pertussis outbreak) to July 31, 2012 (the month after the end of the 2012 respiratory viral season). The respiratory viral season was defined post hoc as the period of time encompassing peak volume of submitted respiratory specimens. All specimens from patients obtained during inpatient and ambulatory visits, including Emergency Department (ED) and Urgent Care facilities, were eligible for inclusion. However, during this time period, the FDA cleared RM-PCR was restricted to testing and reporting of respiratory viruses while LD-PCR is specifically used for the pertussis diagnosis. Post hoc data recovery pertaining to *B. pertussis* detection by RM-PCR was made available through the manufacture upon FDA clearance of the expanded panel in October 2012.

Samples were excluded from analysis if patients had underlying severe immunosuppressive conditions (hematological or oncologic malignancies, or hematopoietic stem cell transplantation), or were in intensive care units (unless they were tested first in the ED) due to frequent repeat testing and active surveillance protocols associated with these patients. Inclusion and exclusion criteria for repeat patient specimens were as follows: (1) for pertussis LD-PCR diagnosis, repeat specimens from the same patient within 4 weeks with identical results (both positive, both negative) were excluded; (2) for respiratory viral diagnosis by RM-PCR, repeat specimen(s) resulting in detection of the same agent(s) in ≤14-day period were excluded; (3) detection of different viruses on repeat specimens from a single patient >14 days apart were considered to reflect different infectious episodes, and each specimen was included independently in the analysis; (4) if both positive and negative viral results were reported from specimens obtained within a 14 day period, only the positive result(s) were included; and (5) if different viruses were detected on repeat specimens within a 14 day period, all viral agents were considered to be from a single sample in the analysis, as were specimens with multiple viruses detected in a single swab.

Pediatricians and primary care physicians were informed by the local health jurisdiction during the state-wide pertussis outbreak period regarding clinical criteria meeting 2010 case definition for pertussis or whooping cough illness published by Centers for Disease Control and Prevention (CDC) [11]. The assessment of physician suspicion of pertussis as a potential diagnosis was based on presence of pertussis laboratory orders for LD-PCR alone by the responsible physician. The assessment of physician suspicion of potentially either a respiratory viral or pertussis infection was based on the presence of physician orders for both pertussis by LD-PCR and respiratory viruses by RM-PCR. We therefore planned to evaluate three groups (or cohorts) in our analysis: (1) children suspected for pertussis alone (with only LD-PCR for pertussis ordered); (2) children in whom pertussis was not suspected (with only RM-PCR for respiratory viruses ordered); and (3) children suspected for viral illness and/or pertussis (with both LD-PCR and RM-PCR ordered).

### Specimens

Deep posterior nasopharyngeal (NP) specimens collected on thin-aluminum wire shafts with small Dacron/Rayon swab tips (Hardy Diagnostics, Santa Maria, CA), kept at 4 °C without transport medium, were required for daily batch testing by LD-PCR. Nylon flocked mid-turbinate (midNT) swabs on a flexible plastic shaft rotated 360° and inoculated in 3-ml Universal Transport Medium (UTM, Copan Diagnostics, Brescia, Italy) were sent promptly for RM-PCR. Two lengths of nylon flocked swabs were used, both with circular collars to ensure standard sampling with indicator for depth: one with a shaft length of 2.5 cm (code 56750CS01) for children ≤2 years or a 5.5 cm long shaft (code 56380CS01) for children >2 years [12]. Samples were typically collected by trained nurses and transported to the laboratory within 1 h.

### LD-PCR for pertussis pathogens

NP swabs were refrigerated if >2 h delay in transport was anticipated. The NP specimen was eluted by vortexing for 20 s in 1 ml saline and concentrated by centrifugation [8]. The LD-PCR was designed to detect and differentiate *B. pertussis*, *B. parapertussis*, and *B. holmesii* based on three species-discriminative DNA targets and melt peak characteristics [8]. In addition, LD-PCR could accommodate up to 10 specimens per 96-well plate with 2.5 h batches feeding into 3–4 thermocyclers during specimen surges [13]. The limit of detection for LD-PCR was determined to show 90 % reproducibility at a minimum of 15 copies per PCR reaction. Consensus criteria for interpreting results used 3 targets, where either 2–3 positive targets with specific melt curve (IS481, *ptx* promoter region, and/or *recA*) or a single reproducible positive target were accepted as positives, with a contingency that if IS481 was the only target positive, test to rule out *B. holmesii* is carried out [8]. Testing was batched; results were typically available within 4–15 h. Bacterial culture for pertussis was performed on all PCR-positive specimens by inoculating the same swab (post PCR elution) on Regan-Lowe agar. A sheep blood agar was added if specimen was positive for *B. parapertussis* or *B. holmesii*. Phenotypic colonies from Regan-Lowe or non-selective blood agar were further speciated and confirmed by LD-PCR.

### RM-PCR for respiratory viruses and pertussis

MidNT swabs in UTM were submitted to the laboratory at room temperature within 1 h [7]. After 20 s of vortex, an aliquot of the solution was added to the RM-PCR card according to manufacturer’s specifications (BioFire). The RM-PCR utilizes a two-step nested multiplex PCR process, including primary multiplex PCR followed by an array of organism-specific second-stage PCR reactions [14]. The RM-PCR for pertussis targets only the toxin promoter region; cross reactivity with *B. parapertussis* is not observed at concentrations less than 10^6^ CFU/ml [15]. Although the limit of detection was listed in the FilmArray product description for *B. pertussis* at 4000 CFU/ml, we have further verified that the sensitivity of the test can approach 31 CFU/ml using serial dilutions running in duplicate. The specific RM-PCR panel at the time was approved to detect 8 viral pathogens and their subtypes, including influenza A (Flu A), influenza B (Flu B), RSV, parainfluenza viruses(PIV) 1–4, human metapneumovirus (HMPV), coronavirus (CoV) HKU1 and NL63, adenovirus (AdV), and rhinovirus/enterovirus (RhV). This assay detected but did not reveal results from three bacterial pathogens (*B. pertussis*, *C. pneumoniae*, and *M. pneumoniae*) until FDA approval in October, 2012.

### Statistical analysis

Proportions were compared using Chi square, McNemar’s exact Chi square, two-sample test of proportions, and one-way ANOVA as appropriate. Statistical analyses were performed using Stata 12.1 (Statacorp, College Station, TX).

## Results

### Specimens

Altogether, 1075 (94.1 %) of 1142 NP specimens tested by LD-PCR met inclusion criteria for analysis while 3566 (64.4 %) of 5523 midNT specimens tested by RM-PCR were included (Table 1; Fig. 1). Patients with NP specimens tested by LD-PCR alone were designated as “Cohort 1” (n = 584; Table 1; Fig. 1). Patients with midNT specimens tested by RM-PCR alone for respiratory viral pathogens was designated as “Cohort 2” (n = 3071; Table 1; Fig. 1). “Cohort 3” was defined as the group of patients on whom both LD-PCR and RM-PCR were ordered within 72 h of each other and are otherwise described as “dual-tested” specimens (n = 490; Table 1; Fig. 1).

**Table 1:** Comparison of viral and *Bordetella* pathogen detection rate among cohorts of patients with different tests ordered by clinicians

	Cohort 1: pertussis PCR alone N = 584 (% positive)	Cohort 2: viral PCR alone N = 3071 (% positive)	Cohort 3: dual tested N = 490 (% positive)	*p* value
Median age	3 years	2 years	0.54 years	<0.001
Age range	6 days–21 years	3 days–21 years	2 days–20 years	NA
Patient age <24 months	240 (41 %)	1268 (41 %)	369 (75 %)	<0.001
*Bordetella* pathogen detection by LD-PCR (%)	n = 21 (3.6 %), including 3 *B. parapertussis* ^a^	NA	n = 22 (4.5 %), including 2 *B. parapertussis* ^a^	0.5
*B. pertussis* detection by RM-PCR (%)	NA	n = 4 (0.1 %)	n = 24 (4.9 %)	<0.001
Combined detection by both RM-PCR and LD-PCR: n (%)	NA	NA	n = 27 (5.5 %), including 2 *B. parapertussis* ^a^	NA
Organism isolation by culture: n (% of culture positive over detection by LD-PCR)	n = 14 (14/21 or 66.7 %), including 2 *B. parapertussis* ^a^	NA	n = 10 (10/22 or 45.5 %), including one *B. parapertussis* ^a^	0.16
Rate of viral positive detection: n (%)	NA	1988 (64.6 %)	367 (74.8 %)	<0.001
Rate of viral positive in patients age < 24 months: n/n (%)	NA	840/1268 (66.2 %)	290/369 (78.6 %)	<0.001
Flu A	NA	164 (5.3)	17 (3.5)	<0.001 (combined Flu A and B rates)
Flu B	NA	360 (11.7)	30 (6.1)	
PIV 1-4	NA	83 (2.7)	33 (6.7)	<0.001
RSV	NA	433 (14.1)	124 (25.3)	<0.001
HMPV	NA	186 (6.0)	35 (7.1)	0.4
AdV	NA	88 (2.9)	17 (3.5)	0.5
RhV	NA	741 (24.1)	143 (29.2)	0.02
CoV	NA	111 (3.6)	24 (4.9)	0.17
2–3 Viruses	NA	170 (5.5)	54 (11.0)	<0.001

^a^n: includes *B. parapertussis*

### Results of pertussis diagnostic assays

The performances of LD-PCR and RM-PCR for detection of pertussis pathogens were compared in the dual-tested group, Cohort 3 (n = 490). A total of 27 (5.5 %) positive specimens were detected in this cohort, including both *B. pertussis* (n = 25) and *B. parapertussis* (n = 2) (Table 1; Fig. 1). If the combined detection of pertussis pathogens (total n = 27, including two *B. parapertussis*) by either RM-PCR or LD-PCR was used as the reference standard, LD-PCR detected 22 of the 27 (81 %) while RM-PCR detected 24 of the 27 (89 %). If excluding the two cases of *B. parapertussis* (a species not included in the RM-PCR panel), LD-PCR detected 20 of the 25 (80 %) *B. pertussis* while RM-PCR detected 24 of the 25 (96 %, p = 0.2 for difference in proportions by McNemar’s exact Chi square). Two of 5 cases of *B. pertussis* missed by LD-PCR were positive for a single target but were not reproducible; the one case missed by RM-PCR was positive for a single target and reproducible by LD-PCR.

Six of the 490 specimens in Cohort 3 had discordant results, with one specimen positive only by LD-PCR only and 5 samples positive by RM-PCR only (Table 1). The median age of these 6 subjects was 1.9 years (range 6 weeks to 10 years), with respiratory symptoms present for a median of 5.5 days prior to testing (range 2–9 days). All six subjects had cough at their clinical assessment, and four of these reported coughing paroxysms. Only one child had a known exposure to pertussis, occurring 7 weeks earlier, and had received post-exposure prophylaxis with a macrolide antibiotic. Three of these six cases (all positive by RM-PCR) had pertussis diagnosed at another facility (method unknown) within 3 days prior to evaluation at our institution.

### Recognition of pertussis by clinicians

We evaluated the ability of clinicians to recognize pertussis by using the tests ordered as a proxy for physician recognition of pertussis (Table 1). Patients in Cohort 1 (n = 584) included those who were strongly suspected to have pertussis, as only LD-PCR was requested. In this cohort, patient age ranged from 6 days to 21 years [median, 3 years, inter-quartile range (IQR) 0.83–7 years], and the rate of pertussis detection was 3.6 %, representing 18 cases of *B. pertussis* and three cases of *B. parapertussis* (Table 1).

Patients in Cohort 2 (n = 3071) had specimens submitted only for respiratory viral pathogen detection, indicating that pertussis was not suspected. In this cohort, patient age ranged from 3 days to 21 years (median, 2 years, IQR 1.08–7 years). *B. pertussis* was detected in only 4 specimens (0.1 %) after obtaining un-blinded RM-PCR results post hoc, a rate significantly different than that in Cohort 1 (p < 0.001 for comparison, Table 1).

Specimens collected in Cohort 3 (n = 490) were from individuals with age range 2 days–20 years (median 0.54 years, IQR 0.16–1.92 years) whose healthcare providers requested both LD-PCR for pertussis and RM-PCR for respiratory viruses. Patients in Cohort 3 tended to be younger than patients in the other two cohorts: the proportion of patients <24 months of age was 41 % in Cohort 1, 41 % in Cohort 2, and 75 % in Cohort 3 (p < 0.001 by Chi square). The detection of pertussis pathogens in Cohort 3 was the highest of all three cohorts (5.5 %), with 2 *B. parapertussis* (Table 1).

### Clinically unsuspected pertussis detection

Four children from Cohort 2 (respiratory viral testing ordered but not pertussis-specific testing) had pertussis detected by RM-PCR (based on data recovered by the manufacture post hoc), including one case previously diagnosed at another institution. The median age of these 4 subjects was 6.6 years (range 6 weeks to 14 years), and the median duration of respiratory symptoms prior to testing was 5 days (range 2–14 days). All four subjects had cough as a clinical finding but coughing paroxysms were not reported, and there was no indication of receipt of macrolides in the medical record.

### Respiratory viral infections

Pertussis and respiratory viral pathogens were present in respiratory specimens in all age groups and all study months (Table 1, Fig. 1). Although the rate of viral infections in Cohort 1 is not known, the overall viral detection rates in Cohorts 2 and 3 were high, at 64.6 and 74.8 % respectively overall (p < 0.001, Table 1), and 66.2 and 78.6 % respectively among those <24 months (p < 0.001, Table 1). Detection rates of specific viruses also differed between Cohorts 2 and 3. In particular, the incidence of influenza (Flu A and Flu B) was higher in Cohort 2 (17.0 vs. 9.6 %, p < 0.001). By contrast, Cohort 3 had a higher incidence of RSV (25.3 vs. 14.1 %), PIV (6.7 vs. 2.7 %), or multiple viruses (11 vs. 5.5 %), all p < 0.001 (Table 1).

### Viral and pertussis co-infections

Viral co-infections were common in specimens with pertussis. Three of four specimens positive for *B. pertussis* in Cohort 2 also had viruses detected—two cases with HMPV and one with Flu B. Sixteen of the 27 (59 %) specimens positive for pertussis pathogens in Cohort 3 had simultaneous detection of viral agents: RhV in 8, CoV in 6, and one each for PIV, HMPV and RSV. Two of 16 specimens contained more than one viral agent—one with RhV and PIV, the other with CoV and HMPV.

### Culture confirmation of pertussis detected by LD-PCR

Bacterial culture was performed in all 43 specimens positive by LD-PCR (21 from Cohort 1 and 22 from Cohort 3). Pertussis pathogens were isolated from 55.8 % of these 43 specimens (n = 24; *B. pertussis* from 21 and *B. parapertussis* from 3 specimens) after 2–15 days of incubation—demonstrating the slow turn-around time and poor diagnostic utility of pertussis culture (Table 1). Rates of isolation of viable organisms were similar (p = 0.16) between Cohort 1 (14/21, 66.6 %) and Cohort 3 (10/22, 45.5 %). None of the 6 specimens with discrepant findings between LD-PCR and RM-PCR had positive culture results.

## Discussion

We assessed the ability of an RM-PCR platform and an LD-PCR test to detect pertussis pathogens in the context of a state-wide pertussis epidemic. Based on the retrospective analysis, the two PCR assays achieved similar sensitivity despite their differences in sampling, collection media, and amplification technology. We also found that clinicians were relatively effective at identifying children at higher risk for pertussis.

In this study, we demonstrated that both midNT specimens collected with nylon flocked swabs and NP specimens collected with Dacron/Rayon swabs were effective for *B. pertussis* detection. These results are similar to findings reported by Esposito et al. [12] and Walsh et al. [16]. Anecdotally, in our center the use of midNT swabs is preferred by health care professionals as the depth of sampling can be standardized with age appropriate visual guides. This approach may in fact promote standardized collection of high quality specimens.

Over the past decade remarkable progress has been made in the development of RM-PCR methods for the concomitant rapid diagnosis of viral and bacterial respiratory infections [7]. Our results suggest similar performance between RM-PCR and LD-PCR methods in identifying pertussis pathogens. Although we were unable to culture pertussis from the 6 samples with discordant results, these cases are likely to be true positives based on evidence of exposure to pertussis, detection of pertussis by other laboratories, and/or the presence of coughing paroxysms. It is possible that the discordant specimens had low copy number of *B. pertussis* DNA and/or sampling variation. The 5 cases missed by LD-PCR could potentially also be related to PCR inhibition with aluminum-shaft swabs [17]. Limitations associated with the design of RM-PCR include the capacity to test only one specimen at a time and to detect only *B. pertussis.* Consequently, the emergence of *B. parapertussis* and *B. holmesii* may be overlooked [13, 18]. In addition, the use of antibiotic-containing media for specimen collection precludes culture for viable organisms, necessitating both molecular and epidemiological investigations [3, 19].

The accuracy of clinical diagnosis of viral versus pertussis infection in children has not been well documented [20]. Clinicians’ abilities to identify patients at risk for pertussis appeared to be relatively effective in our study; pertussis pathogens were detected over 50 times more frequently in dual-tested Cohort 3 (5.5 %) compared to those ordered only for viral testing (Cohort 2, 0.1 %). Influenza appeared to be recognized by clinicians as well; nearly twice as many laboratory-confirmed Flu A/B cases occurred in the viral-only tested Cohort 2 compared to the dual tested Cohort 3 (17 vs. 9.6 %, respectively; p < 0.001). Our retrospective analysis supports the use of RM-PCR in children <24 months as the findings of both respiratory viruses (including multi-viral co-infections) and pertussis are significantly higher in this age group (Table 1).

The implementation of an innovative RM-PCR assay including respiratory viral and bacterial pathogens in a hospital-based laboratory is not without its own challenges. Although clinical assessment can be effective, ordering of a single test that produces multiple results can be problematic. Existing laboratory billing models and its governance have not yet caught up with these multiplex PCR platforms, and the assignment and communication of “unrequested” agents generated by such broad test panels can potentially cause operational challenges and reimbursement difficulties for the laboratory and the institution [13, 21].

Our study was limited by the retrospective design and the lack of complete parallel data for both pertussis and respiratory viruses at every patient visit. We also note that our study occurred during a period of high awareness of pertussis disease. However, the unsolicited test selection and specimen collection by multiple clinicians, combined with the ability to obtain pertussis data from viral test specimens retrospectively, permitted the analysis of physician clinical decision making without study bias.

Our experience during a pertussis epidemic demonstrated the diagnostic acumen of clinicians, the ability to use mid-nasal flocked swabs to detect pertussis without compromise in sensitivity when compared to NP specimens, and the correlation of results from an integrated bacterial-viral RM-PCR assay with a more labor-intensive, well-established, multi-target LD-PCR assay during a specimen surge. The RM-PCR platform has the ability to identify relevant respiratory pathogens quickly, with implications for both optimal treatment and infection prevention management in younger children.
